# Extracellular Polyhydroxyalkanoate Depolymerase by *Acidovorax* sp. DP5

**DOI:** 10.1155/2015/212159

**Published:** 2015-11-17

**Authors:** S. Vigneswari, T. S. Lee, Kesaven Bhubalan, A. A. Amirul

**Affiliations:** ^1^Malaysian Institute of Pharmaceuticals and Nutraceuticals, NIBM, MOSTI, 11700 Penang, Malaysia; ^2^Institute of Marine Biotechnology, Universiti Malaysia Terengganu, 21030 Kuala Terengganu, Malaysia; ^3^School of Biological Sciences, Universiti Sains Malaysia, 11800 Penang, Malaysia; ^4^School of Marine and Environmental Science, Universiti Malaysia Terengganu, 21030 Kuala Terengganu, Malaysia; ^5^Centre for Chemical Biology, Universiti Sains Malaysia, 11900 Penang, Malaysia

## Abstract

Bacteria capable of degrading polyhydroxyalkanoates (PHA) by secreting extracellular depolymerase enzymes were isolated from water and soil samples collected from various environments in Malaysia. A total of 8 potential degraders exhibited clear zones on poly(3-hydroxybutyrate) [P(3HB)] based agar, indicating the presence of extracellular PHA depolymerase. Among the isolates, DP5 exhibited the largest clearing zone with a degradation index of 6.0. The highest degradation activity of P(3HB) was also observed with depolymerase enzyme of DP5 in mineral salt medium containing P(3HB). Based on biochemical characterization and 16S rRNA cloning and sequencing, isolate DP5 was found to belong to the genus *Acidovorax* and subsequently named as *Acidovorax* sp. DP5. The highest extracellular depolymerase enzyme activity was achieved when 0.25% (w/v) of P(3HB) and 1 g/L of urea were used as carbon and nitrogen source, respectively, in the culture media. The most suitable assay condition of the depolymerase enzyme in response to pH and temperature was tested. The depolymerase produced by strain *Acidovorax* sp. DP5 showed high percentage of degradation with P(3HB) films in an alkaline condition with pH 9 and at a temperature of 40°C.

## 1. Introduction

Polyhydroxyalkanoate (PHA), a biodegradable polymer with properties resembling common nondegradable petrochemical based polymer, is being considered as a promising building block for plastic-like materials. Gradual replacement of nondegradable plastics with eco-friendly biodegradable materials will help to reduce plastic waste accumulation in our fragile environment. PHA is accumulated intracellularly as energy-reserve granules. PHA production is initiated by nutrient limiting conditions (e.g., nitrogen or phosphorus) and excess supply of carbon source [1]. Poly(3-hydroxybutyrate) [P(3HB)] is the most commonly occurring PHA in the natural environment; hence, it is commonly used in PHA related studies. PHA is completely degradable upon disposal into the environments [2–5]. PHA does not require special environmental conditions to be degraded as it can undergo the biodegradation process both aerobically and anaerobically [6]. The degradability of biodegradable plastics depends on the degrading organisms available in the environment. The ability of microorganisms to degrade PHA in the environment is mainly dependent on the secretion of specific extracellular PHA depolymerases [7].

To date, about 30 PHA depolymerases with experimentally validated depolymerase activity have been described [8]. The most common types of bacteria associated with depolymerase enzyme study belong to the genera* Cupriavidus* [9, 10],* Alcaligenes* [11],* Comamonas* [12, 13], and* Pseudomonas* [6, 14, 15]. However, limited report is available on the extracellular PHA depolymerase enzyme belonging to* Acidovorax* [16]. Identification of these bacteria suggests that occurrence of extracellular PHA depolymerases is ubiquitous in the environment. Most of the purified depolymerases were specific for short-chain-length PHA (PHA_SCL_) such as 3-hydroxybutyrate (3HB), 3-hydroxyvalerate (3HV), or 4-hydroxybutyrate (4HB). Some examples of well-studied depolymerases belong to* A. faecalis*,* Comamonas *sp., or* P. lemoigne* [17–19].

Generally, there are two types of PHA depolymerase which can be classified as intracellular or extracellular based on their mode of reaction towards the substrates [20]. PHA-degrading bacteria secrete extracellular PHA depolymerases in order to hydrolyze PHA into water-soluble intermediates which are then used as carbon and energy sources for growth [21, 22]. In the natural environment, PHA granules accumulated in bacteria are released into the environment upon cell lysis. Some PHA producing bacteria are also known to mobilize PHA (intracellular degradation) in stressed or unbalanced environmental conditions for survival.

The factor that influences the rate of biodegradation of PHA includes the physical properties of the polymer, environmental conditions such as temperature and pH, and the microbial population in a given environment [23]. To date, there are only few reports on the isolation and identification of such microbes found in Malaysian environment. This study reports the occurrence of PHA-degrading bacteria in different environments in Malaysia. The PHA-degraders were isolated and tested for the production of extracellular PHA depolymerase enzyme. The depolymerase enzyme was tested for degradation on P(3HB) film and factors affecting its activity such as pH and temperature were investigated.

## 2. Materials and Methods

### 2.1. Growth and Enzyme Production Media

The chemicals used in this study are of analytical grade (Merck, Sigma, Technical Grade AR, R&M Chemicals and Oxoid) and used according to the manufacturers' protocols. Mineral salt medium supplemented with P(3HB) [MSM-P(3HB)] was used in this study for bacterial growth and enzyme production. The MSM consists of 2.56 g/L K_2_HPO_4_ (R&M Chemicals), 2.08 g/L KH_2_PO_4_ (R&M Chemicals), and one of the following selected nitrogen sources (1 g/L) such as ammonium chloride (NH_4_Cl), ammonium sulphate (NH_4_SO_4_), ammonium nitrate (NH_4_NO_3_), diammonium hydrogen phosphate [(NH_4_)_2_HPO_4_], and urea [(NH_2_)_2_CO] (R&M Chemicals) as the main components with pH adjusted to 6.8. To this solution, 0.75% (w/v) of P(3HB) powdered granules were added and the solution was sterilized by autoclaving at 121°C for 15 min under 15 psi pressure (103 kPa). During inoculation, filter sterilized 0.5 g/L MgSO_4_ (R&M Chemicals), 500 *μ*g/L C_6_H_8_FeNO_7_ (R&M Chemicals), 0.1 *μ*g/L CaCl_2_ (R&M Chemicals), and 0.2 g/L (CH_3_COONa)_2_·6H_2_O (R&M Chemicals) [pH 6.0] were added. Solid media were prepared by the addition of 2.0% (w/v) bacteriological agar powder.

### 2.2. Isolation and Identification of PHA-Degrading Bacteria

Soil and water samples collected from different locations surrounding Peninsular Malaysia were screened for PHA-degrading bacteria. The environmental samples were first cultured in MSM-P(3HB) liquid media at 30°C at 200 rpm for 24–48 h. Later, the cultures were diluted in sterile distilled water [24] and plated on MSM-P(3HB) agar and were incubated at 30°C for 5 days. Colonies that exhibited clear zone around them were screened out as PHA-degrading bacteria. Cell growth was monitored by measuring the optical density (OD) [540 nm] at different time intervals [25]. Identification of the isolate was performed based on morphological observation and 16S rRNA cloning and DNA sequencing. The 16S rRNA analysis was carried out by extracting the genomic DNA using GF-1 Bacterial DNA Extraction Kit (Qiagen). Amplification of the 16S rRNA region cloning from extracted bacterial DNA was performed using the following pair of universal primers: 5′—GTGCCAGCMGCCGCGGTAA—3′ and 5′—GGGCCCCGYCAATTCCTTTGARTTT—3′. Purification and concentration of the PCR product was carried out using GF-1 Gel DNA Recovery Kit (Qiagen). Purified PCR products were sequenced and the sequence obtained was screened in GeneBank (NCBI) database for matching homology. Sequence alignment and construction of phylogenetic tree were performed to conclude the identity of the isolate DP5.

### 2.3. PHA Depolymerase Enzyme Assay

The activity of depolymerase was determined based on the method described previously [26]. The standard assay mixture contains 400 mg/mL P(3HB) polymers and 1 mM CaCl_2_ in 50 mM Tris-HCL buffer (pH 7.5). The purified P(3HB) granules (stable suspension) were suspended in the buffer and dispersed using a sonic oscillator (20 kHz, 250 W) for 10 mins. The reaction started by the addition of enzyme which brings about the decrease in turbidity of P(3HB) polymer suspension, which was measured at 650 nm and 37°C, using 1 cm light-path cuvettes. Enzyme activity was calculated using the formula below:

(1) Enzyme  activity  mg/mL/min=change  in  OD650 nm×constant  0.82enzyme  volume×assay  time.

One unit of depolymerase enzyme activity (mg/mL/min) is defined as mg of P(3HB) degraded per mL of enzyme per minute.

### 2.4. P(3HB) Film Preparation

P(3HB) film was prepared by dissolving 0.75 g of the extracted P(3HB) polymer in 20 mL of chloroform and poured into a glass Petri dish (diameter of 9 cm) as a casting surface. The films were then air-dried (24 h) and later vacuum-dried (48 h) to remove remaining solvent. The thickness on the P(3HB) film obtained was 0.06 mm and was used in the enzymatic degradation test by extracellular depolymerase enzyme.

### 2.5. Enzymatic Degradation of P(3HB) Films

Cast films were prepared and cut into 10 mm × 10 mm pieces. The film pieces were then immersed in 40 mL glycine-NaOH buffer solution containing 10 mL (0.05 mg/mL/min) of depolymerase enzyme from* Acidovorax* sp. DP5 in a 250 mL conical flask. The reaction mixture was incubated at 37°C (temperature was varied for degradation experiment in which temperature was a factor) in an incubator shaker at 180 rpm. At each interval of incubation, the films were removed, rinsed with water, and dried to constant weight [27].

## 3. Results and Discussion

### 3.1. Isolation of PHA-Degrading Bacteria from Various Environments

**Figure 1:**
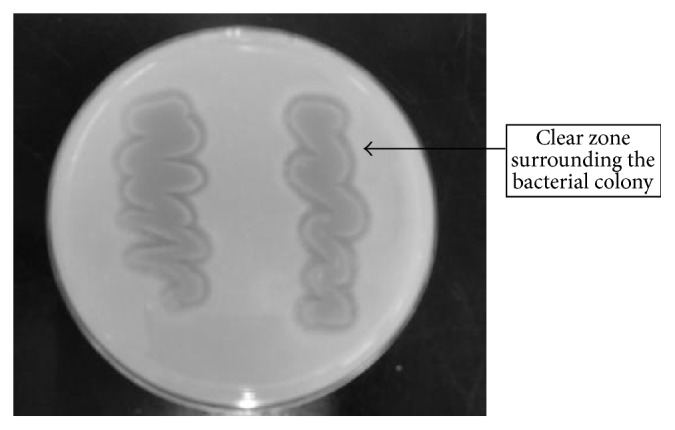
Clear zone formation produced by* Acidovorax* sp. DP5 on P(3HB) agar plate. Cells were grown for 4-5 days at 30°C to form clear zone surrounding the bacterial colony.

It has been estimated that the percentage of PHA-degrading microorganisms in the environment falls in the range of 0.5–9.6% of the total colonies present [28]. In this study, a total of 18 strains were isolated by culturing environmental samples in MSM-P(3HB) broth. This method serves as an initial screening for bacteria capable of hydrolyzing P(3HB) using it as carbon source for growth. Next, the isolates were further screened on MSM-P(3HB) agar to avoid the presence of pseudo-positive degraders. This is because it is possible for other non-P(3HB) degrading bacteria to establish symbiotic reactions in the presence of positive P(3HB) degraders. Formation of “clear-zones” around bacterial colonies indicates positive results and confirms the P(3HB) degrading ability of strains [29]. From the 18 isolates initially obtained, only eight were identified as potential PHA-degrading microorganisms based on the formation of clear zone (Figure 1) and by observing the degradation index. The formation of the clear zones around the colonies is an indication that the polymer is hydrolyzed by the enzyme into water-soluble products [29]. In order to be classified as PHA-degraders, a degradation index of more than one should be obtained. It was visually observed and calculated that eight strains out of the 18 isolates produced a degradation index of more than 1.00.

**Figure 2:**
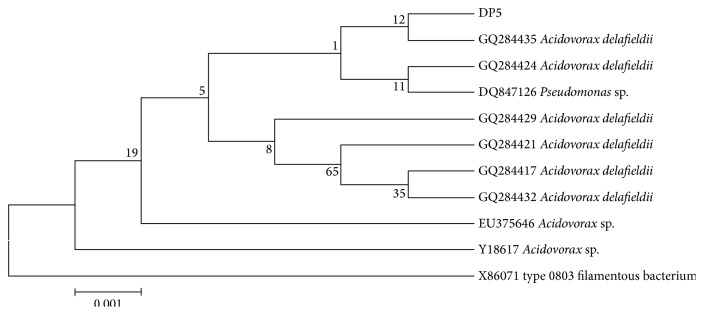
Neighbour-joining phylogenetic tree of* Acidovorax* sp. DP5 and related bacteria based on 16S rRNA sequence comparisons. Accession numbers are given.

The result obtained (Table 1) showed that the microorganism coded as DP5 gave the highest degradation index of 6.00 followed by DP10 with the degradation index of 5.00. In order to determine the ability in producing P(3HB) degrading enzyme, enzyme assay of the 8 isolates was studied by using the samples at 48 h growth phase. Based on the enzyme activity assay, the DP5 bacterium showed the highest activity which was 0.035 mg/mL/min compared to the other isolates (Table 2). DP5 was found to exhibit the highest degradation index as well as depolymerase activity. DP5 was identified as Gram negative and rod shaped (Table 3). The 16S rRNA sequence of strain DP5 exhibited 99% similarity to* Acidovorax delafieldii*. Therefore, the strain was denoted as* Acidovorax* sp. DP5. The phylogenetic comparison of strain* Acidovorax* sp. DP5 within its genus is shown in Figure 2. In a separate study, Feng et al. reported the ability of* Acidovorax* sp. TP4 to degrade P(3HB) homopolymer and copolymer containing 3-hydroxyvalerate [30]. Salim et al. reported the presence of* Acidovorax* sp. and* Ralstonia* sp. from lake water and soil samples [24]. Based on Volovaa and coworkers, PHA-degrading* Enterobacter* sp.,* Bacillus* sp., and* Gracilibacillus* sp. strains were isolated from tropical marine water [31].

### 3.2. Growth and Depolymerase Enzyme Activity

**Figure 3:**
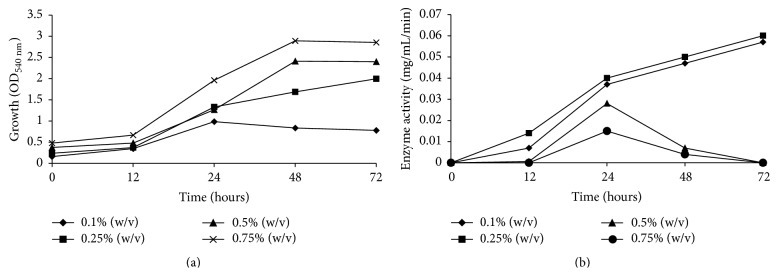
Effect of P(3HB) concentration (w/v) on the (a) bacterial growth and (b) enzyme activity. Values are mean of two replicates.

Growth of* Acidovorax* sp. DP5 and its depolymerase enzyme activity was further evaluated by manipulating the concentration of P(3HB) and nitrogen source. DP5 was cultivated in MSM containing different concentration of P(3HB) [0.1%–0.75% w/v] as the sole carbon source. Based on the result obtained in Figure 3(a), maximum growth was obtained at the highest concentration of P(3HB). It was clearly observed that the growth of* Acidovorax* sp. DP5 was proportional with concentration of P(3HB). At P(3HB) concentration of 0.75%, the growth of cells increased almost 4-fold from 12 to 48 h of cultivation. The growth of DP5 was almost reaching stationary phase after 72 h. In contrary, it was found that feeding of a low concentration of P(3HB) as substrate favored the high activity of depolymerase enzyme (Figure 3(b)).

**Figure 4:**
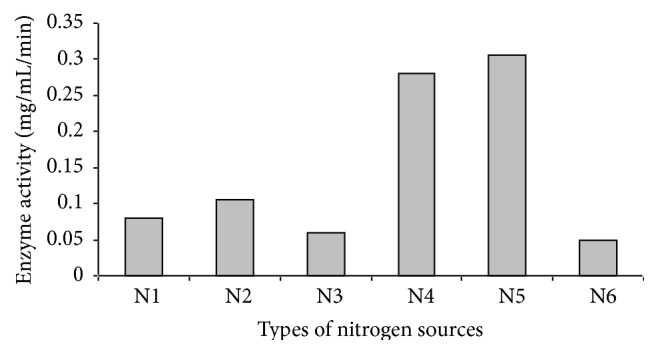
Effect of various nitrogen sources on the enzyme activity. N1: NH_4_Cl, N2: (NH_4_)_2_SO_4_, N3: NH_4_NO_3_, N4: (NH_4_)_2_HPO_4_, N5: (NH_2_)_2_CO, and N6: control (without nitrogen source). Values are mean of two replicates.

The highest enzyme activities of 0.056 mg/mL/min and 0.066 mg/mL/min were achieved at 72 h of incubation when P(3HB) concentration was 0.1% and 0.25% (w/v), respectively. On the other hand, no enzymatic activity was detected at 72 h with cultures fed with 0.5% (w/v) and 0.75% (w/v) of P(3HB). The depolymerase activity showed a sharp decrease for these concentrations of P(3HB) after 24 h of cultivation. This result might contradict the usual presumption that a higher P(3HB) concentration would possibly contribute to a higher depolymerase enzyme activity. The reduction in enzyme activity may have been caused by surplus product formation when higher concentrations of P(3HB) were supplied. This was evident in increased cell growth observed with higher P(3HB) concentrations (Figure 3(a)). Nitrogen plays a crucial role in supporting bacterial growth and the type of nitrogen source supplied in the medium is known to affect the secretion of enzymes and production of an intermediate and the final product. Various nitrogen sources such as NH_4_Cl, NH_4_SO_4_, NH_4_NO_3_, (NH_4_)_2_HPO_4_, and (NH_2_)_2_CO [1 g/L] were screened in relation to depolymerase enzyme activity. Among these nitrogen sources, urea resulted in the highest enzyme activity (Figure 4).

### 3.3. Enzymatic Degradation of P(3HB) Film

The enzymatic degradation of P(3HB) films is known to take place via two steps, adsorption and hydrolysis [32]. The coupled actions of adsorption toward the P(3HB) polymer chains on the surface of the film by the binding domain of depolymerase and the cleavage of polymer chain result in the breakdown of P(3HB) polymer chains to oligomers [33]. PHA polymers can be categorized into native and denatured PHA granules. The native PHA granules which contain lipids and proteins are rapidly hydrolyzed by intracellular PHA depolymerase [8]. On the other hand, the partially crystalline denatured granules are degraded by extracellular depolymerase [8].

**Figure 5:**
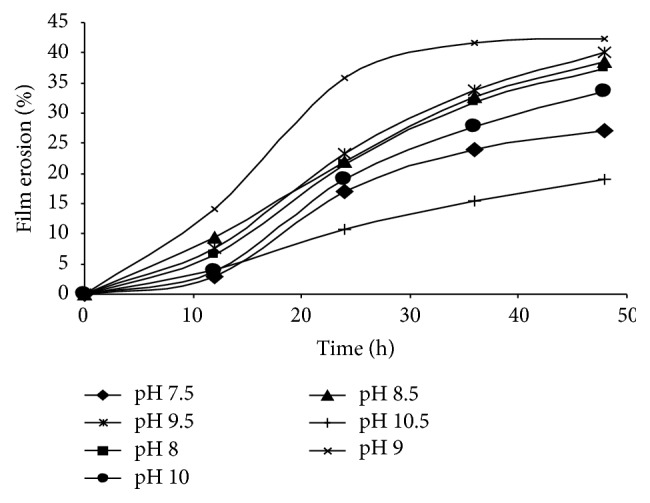
Effect of various pH values on the degradation of P(3HB) films in an aqueous solution of depolymerase at 37°C. Values are mean of two replicates.

Besides microbial population and the properties of the plastic materials, surrounding temperature and pH have been found to have a strong influence on the rate of biodegradation of PHA polymers. It was previously reported that the optimum activity of depolymerase enzyme was achieved in alkaline conditions [22]. In this study, the effect of various pH values in the alkaline region ranging from 7.5 to 10.5 towards the degradation process of P(3HB) films was evaluated. Varying pH conditions revealed different surface erosion of P(3HB) films by the extracellular depolymerase of DP5 microorganism. Based on results obtained in Figure 5, pH 9 gave the highest percentage of degradation up to 42% of P(3HB) film. At this pH, the degradation reached around 35% at 24 h, whereas, with other pH values, the degradation was only in the range of 10–25%. It was observed that the degradation of P(3HB) film was the lowest at pH 10.5. Hence, it could be concluded that the most suitable pH required by depolymerase for maximum activity is at 9.

**Figure 6:**
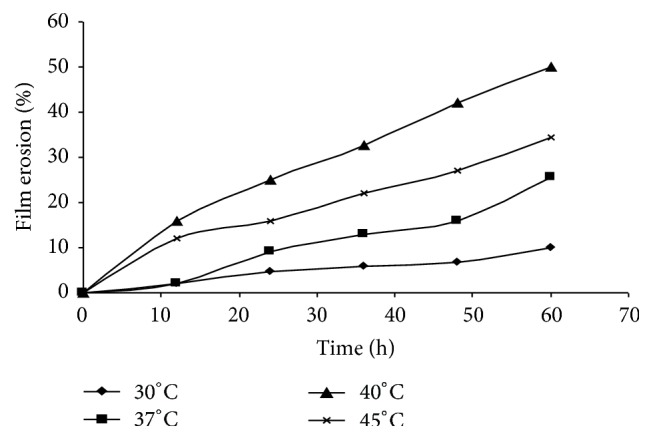
Effect of percentage of weight loss (degradation) of P(3HB) films at various temperatures at pH 9. Values are mean of two replicates.

On the other hand, temperature also has a significant influence on the solubility of organic compounds [20]. At a high temperature, P(3HB) was considered to be amorphous and more sensitive to the degrading enzyme, allowing its degradation. Based on result obtained in Figure 6, it was found that, by increasing the incubation temperature from 30°C to 40°C, the surface erosion of the film can be increased up to 50% at 60 h of cultivation. Degradation was the highest at 40°C, whereby up to 25% of film erosion was observed within 24 h. However, for the other temperatures, the percentage of degradation was below 20% at this point of time. Further increment in temperature only reduced the overall rate of degradation. From these experiments, it was concluded that pH 9 and temperature of 40°C were the optimal conditions for competent hydrolysis of P(3HB) film using the depolymerase enzyme secreted by* Acidovorax* sp. DP5. The molecular weights and mechanical properties of enzyme degraded P(3HB-*co*-4HB) copolymer scaffolds using the optimal conditions were also studied (Table 4). Interestingly, it was observed that the 4HB mol% increased by 2–8 mol% when the copolymer films were degraded with the depolymerase enzyme.

## 4. Conclusion

*Acidovorax* sp. DP5 isolated from the Malaysian environment was found capable of competently hydrolyzing P(3HB). The depolymerase enzyme produced by this strain showed high percentage of degradation with P(3HB) film in an alkaline condition with pH 9 and at temperature of 40°C. The findings of this research indicate the possibilities of further exploiting the use of this depolymerase enzyme in enzyme kinetics studies and its application in PHA-degradation experiments.

## Figures and Tables

**Table 1:** The degradation index based on the formation of clear zone on MSM-P(3HB) agar^a^.

Isolates	Clear zone diameter (*x*) (cm)	Colony diameter (*y*) (cm)	DI^b^ (*x*/*y*)
DP1	3.80	1.40	2.71
DP2	3.00	0.80	3.75
DP3	2.30	1.00	2.30
DP4	3.85	1.55	2.48
DP5	4.50	0.75	6.00
DP8	3.75	1.50	2.50
DP10	4.45	0.80	5.56
DP12	2.20	2.10	1.05

^a^Cells were grown for 4-5 days at 30°C before the clear zone was formed. Values are mean of two replicates.

^b^Degradation index (DI) is calculated based on clear zone diameter/diameter of colony.

**Table 2:** Comparison of the depolymerase enzyme activity assays of the eight isolates.

Isolates	Enzyme activity^a^ (mg/mL/min)
DP1	0.032
DP2	ND
DP3	ND
DP4	0.032
DP5	0.035
DP8	0.002
DP10	ND
DP12	0.009

^a^Depolymerase enzyme assayed by growing the bacterium in MSM-P(3HB) broth.

Values are mean of two replicates.

ND: not determined, as the enzyme activity was too low to be quantified.

**Table 3:** Taxonomic characteristics of the isolate DP5^a^.

Characteristic	Presence/reactions^b^
Morphological characteristics	
Shape	Rod
Motility	+
Gram staining	−
Features of colonies	
Shape	Circular
Opacity	Opaque
Elevation	Convex
Edge	Entire
Color	White
Physiological characteristics	
Catalase	+
Oxidase	+
Urease	−
Arginine dihydrolase	−
Reduction of nitrate (NO_3_→ NO_2_)	+
Hydrolysis of gelatine	−
Hydrolysis of esculin	−
Nutritional characteristics	
Glucose	+
Arabinose	+
Mannose	+
Mannitol	+
N-Acetyl-glucosamine	−
Maltose	−
Gluconate	+
Caprate	−
Adipate	+
Malate	+
Citrate	−

^a^Morphological characteristics and features of the isolate were monitored on P(3HB) agar plate. Physiological and nutritional characteristics were determined using API 20 NE.

^b^+ indicates a positive result; − indicates a negative result.

**Table 4:** Molecular weights and mechanical properties of enzyme degraded P(3HB-*co*-4HB) copolymer scaffolds.

Polymer	4HB composition (mol%)^a^	*M*_*n*_^b^ ×10^3^ Da	*M*_*w*_/*M*_*n*_^b^	Glass transition temperature^c^ °C (*T*_*g*_)	Melting temperature^c^ °C (*T*_*m*_)	Tensile strength (MPa)^d^	Elongation at break (%)^d^
P(3HB) (control)	—	66 ± 6	1.8 ± 0.1	5 ± 1	161 ± 1	—	—
P(3HB-*co*-16%4HB)	21 ± 2	75 ± 5	2.1 ± 0.3	11 ± 3	156 ± 3	0.31 ± 0.15	48 ± 14
P(3HB-*co*-29%4HB)	33 ± 4	68 ± 6	1.7 ± 0.2	15 ± 2	154 ± 2	0.12 ± 0.06	7 ± 1
P(3HB-*co*-45%4HB)	49 ± 2	60 ± 4	1.8 ± 0.2	14 ± 2	151 ± 2	0.32 ± 0.07	30 ± 16
P(3HB-*co*-63%4HB)	71 ± 3	61 ± 10	2.4 ± 0.3	10 ± 2	136 ± 2	0.42 ± 0.21	15 ± 5
P(3HB-*co*-91%4HB)	93 ± 1	49 ± 7	1.3 ± 0.7	32 ± 3	45 ± 3	0.25 ± 0.05	31 ± 5

^a^Calculated from GC analysis; changes in 4HB monomer composition of scaffolds subjected to enzymatic degradation.

^b^Calculated from GPC analysis; *M*_*n*_: number-average molecular weight; *M*_*w*_/*M*_*n*_: polydispersity index.

^c^Calculated from DSC analysis.

^d^Determined using GoTech A1-3000 Tensile Machine.

## References

[1] Kachrimanidou V., Kopsahelis N., Papanikolaou S. (2014). Sunflower-based biorefinery: poly(3-hydroxybutyrate) and poly(3-hydroxybutyrate-*co*-3-hydroxyvalerate) production from crude glycerol, sunflower meal and levulinic acid. *Bioresource Technology*.

[2] Brandl H., Püchner P. (1991). Biodegradation of plastic bottles made from ‘Biopol’ in an aquatic ecosystem under in situ conditions. *Biodegradation*.

[3] Byrom D. (1993). The synthesis and *biodegradation* of polyhydroxyalkanoates from bacteria. *International Biodeterioration and Biodegradation*.

[4] Doi Y. (1990). *Microbial Polyesters*.

[5] Steinbüchel A., Valentin H. E. (1995). Diversity of bacterial polyhydroxyalkanoic acids. *FEMS Microbiology Letters*.

[6] Calabia B. P., Tokiwa Y. (2004). Microbial degradation of poly(D-3-hydroxybutyrate) by a new thermophilic Streptomyces isolate. *Biotechnology Letters*.

[7] Verlinden R. A. J., Hill D. J., Kenward M. A., Williams C. D., Radecka I. (2007). Bacterial synthesis of biodegradable polyhydroxyalkanoates. *Journal of Applied Microbiology*.

[8] Knoll M., Hamm T. M., Wagner F., Martinez V., Pleiss J. (2009). The PHA depolymerase engineering database: a systematic analysis tool for the diverse family of polyhydroxyalkanoate (PHA) depolymerases. *BMC Bioinformatics*.

[9] Jendrossek D. (2002). Extracellular polyhydroxyalkanoate (PHA) depolymerases: the key enzymes of PHA degradation. *Biopolymers Online*.

[10] Saito T., Suzuki K., Yamamoto J. (1989). Cloning, nucleotide sequence, and expression in *Escherichia coli* of the gene for poly(3-hydroxybutyrate) depolymerase from *Alcaligenes faecalis*. *Journal of Bacteriology*.

[11] Bachmann B. M., Seebach D. (1999). Investigation of the enzymatic cleavage of diastereomeric oligo(3-hydroxybutanoates) containing two to eight HB units. A model for the stereoselectivity of PHB depolymerase from *Alcaligenes faecalis* T_1_. *Macromolecules*.

[12] Jendrossek D. (1998). Microbial degradation of polyesters: a review on extracellular poly(hydroxyalkanoic acid) depolymerases. *Polymer Degradation and Stability*.

[13] Kasuya K.-I., Doi Y., Yao T. (1994). Enzymatic degradation of poly[(R)-3-hydroxybutyrate] by *Comamonas testosteroni* ATSU of soil bacterium. *Polymer Degradation and Stability*.

[14] Mukai K., Yamada K., Doi Y. (1994). Efficient hydrolysis of polyhydroxyalkanoates by *Pseudomonas stutzeri* YM1414 isolated from lake water. *Polymer Degradation and Stability*.

[15] Schöber U., Thiel C., Jendrossek D. (2000). Poly(3-hydroxyvalerate) depolymerase of *Pseudomonas lemoignei*. *Applied and Environmental Microbiology*.

[16] Kobayashi T., Sugiyama A., Kawase Y., Saito T., Mergaert J., Swings J. (1999). Biochemical and genetic characterization of an extracellular poly(3-hydroxybutyrate) depolymerase from *Acidovorax* sp. strain TP4. *Journal of Environmental Polymer Degradation*.

[17] Jendrossek D., Knoke I., Habibian R. B., Steinbuchel A., Schlegel H. G. (1993). Degradation of poly(3-hydroxybutyrate), PHB, by bacteria and purification of a novel PHB depolymerase from *Comamonas* sp.. *Journal of Environmental Polymer Degradation*.

[18] Müller R., Antranikian G., Maloney S., Sharp R. (1998). Thermophilic degradation of environmental pollutants. *Biotechnology of Extremophiles*.

[19] Saito T., Tomita K., Juni K., Ooba K. (1991). In vivo and in vitro degradation of poly(3-hydroxybutyrate) in pat. *Biomaterials*.

[20] Tokiwa Y., Calabia B. P. (2007). Biodegradability and biodegradation of polyesters. *Journal of Polymers and the Environment*.

[21] Jendrossek D., Handrick R. (2002). Microbial degradation of polyhydroxyalkanoates. *Annual Review of Microbiology*.

[22] Jendrossek D., Schirmer A., Schlegel H. G. (1996). Biodegradation of polyhydroxyalkanoic acids. *Applied Microbiology and Biotechnology*.

[23] Mukai K., Yamada K., Doi Y. (1993). Enzymatic degradation of poly(hydroxyalkanoates) by a marine bacterium. *Polymer Degradation and Stability*.

[24] Salim Y. S., Sharon A., Vigneswari S., Mohamad Ibrahim M. N., Amirul A. A. (2012). Environmental degradation of microbial polyhydroxyalkanoates and oil palm-based composites. *Applied Biochemistry and Biotechnology*.

[25] Amirul A. A., Yahya A. R. M., Sudesh K., Azizan M. N. M., Majid M. I. A. (2008). Biosynthesis of poly(3-hydroxybutyrate-*co*-4-hydroxybutyrate) copolymer by *Cupriavidus* sp. USMAA1020 isolated from Lake Kulim, Malaysia. *Bioresource Technology*.

[26] Ohura T., Aoyagi Y., Takagi K.-I., Yoshida Y., Kasuya K.-I., Doi Y. (1999). Biodegradation of poly(3-hydroxyalkanoic acids) fibers and isolation of poly(3-hydroxybutyric acid)-degrading microorganisms under aquatic environments. *Polymer Degradation and Stability*.

[27] Vigneswari S., Vijaya S., Majid M. I. A. (2009). Enhanced production of poly(3-hydroxybutyrate-co-4-hydroxybutyrate) copolymer with manipulated variables and its properties. *Journal of Industrial Microbiology and Biotechnology*.

[28] Suyama T., Tokiwa Y., Ouichanpagdee P., Kanagawa T., Kamagata Y. (1998). Phylogenetic affiliation of soil bacteria that degrade aliphatic polyesters available commercially as biodegradable plastics. *Applied and Environmental Microbiology*.

[29] Nishida H., Suzuki S., Tokiwa Y. (1998). Distribution of poly(*β*-propiolactone) aerobic degrading microorganisms in different environments. *Journal of Environmental Polymer Degradation*.

[30] Feng L. D., Wang Y., Inagawa Y. (2004). Enzymatic degradation behavior of comonomer compositionally fractionated bacterial poly(3-hydroxybutyrate-*co*-3-hydroxyvalerate)s by poly(3-hydroxyalkanoate) depolymerases isolated from *Ralstonia pickettii* T1 and *Acidovorax* sp. TP4. *Polymer Degradation and Stability*.

[31] Volovaa T. G., Boyandina A. N., Vasilievc A. D. (2010). Biodegradation of polyhydroxyalkanoates (PHAs) in tropical coastal waters and identification of PHA-degrading bacteria. *Polymer Degradation and Stability*.

[32] Lucas N., Bienaime C., Belloy C., Queneudec M., Silvestre F., Nava-Saucedo J.-E. (2008). Polymer biodegradation: mechanisms and estimation techniques—a review. *Chemosphere*.

[33] Doi Y., Mukai K., Kasuya K., Yamada K. (1994). *Biodegradable Plastics and Polymers*.

